# Head-to-head comparative study of [^68^Ga]FAPI-04 PET/CT and [^18^F]FDG PET/CT in assessing bone metastasis of various cancers: a systematic review and meta-analysis

**DOI:** 10.3389/fonc.2026.1730986

**Published:** 2026-01-30

**Authors:** MingLiu He, Jian Tan

**Affiliations:** Dazhou Dachuan District People’s Hospital, (Dazhou Third People’s Hospital), Dazhou, China

**Keywords:** bone metastasis, FAPI, FDG, meta-analysis, PET/CT

## Abstract

**Purpose:**

The objective of our systematic review and meta-analysis is to compare the performance of [^68^Ga]FAPI-04 PET/CT and [^18^F]FDG PET/CT in diagnosing bone metastasis of various cancer types. This analysis aims to provide an objective assessment of the diagnostic capabilities of these two imaging modalities.

**Methods:**

We conducted a search on Embase, Web of Science, Pubmed for articles published between 2023 and 2025 that meet our criteria. This study focuses on exploring the diagnostic value of [^68^Ga]FAPI-04 PET/CT and [^18^F]FDG PET/CT in various cancer bone metastases. we used random-effects analysis to assess the diagnostic capabilities of [^68^Ga]FAPI-04 PET/CT and [^18^F]FDG PET/CT in patient-based analysis (PB) with bone metastasis and bone metastatic lesion-based analysis (LB).Heterogeneity of the data was assessed through sensitivity analysis. Furthermore, the included studies underwent quality assessment using the QUADAS-2 tool, which is a bias assessment tool utilized for diagnostic test accuracy studies.

**Results:**

In the initial search, 235 articles were identified, and finally 10 clinical studies were included by excluding related articles. All included studies were retrospective, involved diverse cancer types, and did not contain overlapping patient cohorts from the same institution. In the patient-based analysis (PB), the detection rate of [^68^Ga]FAPI-04 PET/CT was determined to be 0.99 (95% CI: 0.90–1.00, I²=36.5%), whereas that of [^18^F]FDG PET/CT was 0.79 (95% CI: 0.54–0.79, I²=62.4%),Sensitivity analysis revealed potential sources of heterogeneity. In the lesion-based analysis (LB), the detection rate of [^68^Ga]FAPI-04 PET/CT was determined to be 1.00 (95% CI: 0.98–1.00, I²=91.5%), while [^18^F]FDG PET/CT showed a detection rate of 0.71 (95% CI: 0.61–0.80, I²=95.3%), with no significant sources of heterogeneity identified.

**Conclusion:**

By comparing the available data, [^68^Ga]FAPI-04 PET/CT appears to show a higher detection rate for bone metastases in various malignancies compared with [^18^F]FDG PET/CT. However, this finding should be interpreted with caution, as pooled sensitivity, specificity, PPV, NPV, DOR, or HSROC analyses could not be performed due to insufficient available data,

## Introduction

Bone metastasis is a common complication of advanced cancer (1), especially tumors originating from breast and prostate. About 70% of breast cancer and prostate patients have bone metastasis. Unfortunately, once the tumor has bone metastasis, it is extremely prone to fracture (2, 3), bone pain and other complications, the patients’ quality of life is significantly affected (4, 5). Therefore, the accurate diagnosis of malignant tumor bone metastasis plays a crucial role in guiding subsequent treatment and assessing the prognosis of patients. Currently, the use of whole-body scanning with [99mTc]Tc-MDP is considered the standard of practice for detecting bone metastases in malignant tumors. The tracer is absorbed, and the absorption intensity is associated with local blood flow and osteoblast activity. [99mTc]Tc-MDP identifies affected bone cells rather than the tumor itself. This technique relies on the osteoblastic response, thus being highly sensitive for advanced-stage malignant tumor bone metastasis. However, its accuracy in early diagnosis and treatment is not high, and its sensitivity and specificity are not ideal (6).

The [^18^F]FDG PET/CT technology is a PET/CT that combines positron emission tomography (PET) with 18F-fluorodeoxyglucose (FDG) and computed tomography (CT) (7). By imaging the metabolism of glucose in tissue, it provides a non-invasive method for bone metastasis of cancer and provides relevant data for the staging and typing of bone metastatic tumors (8). The European Organization for Cancer Research and treatment (EORTC) imaging team assessed the ability of current mainstream imaging examinations to assess bone metastasis, and [^18^F]FDG PET/CT showed great advantages in terms of whole body coverage, reproducibility, and ease of use in multicenter trials (9). At present, [^18^F]FDG PET/CT is mainly used to distinguish between benign and malignant tumors in multiple organ systems, but its role in the evaluation of bone tumors is not clear (10). Fibroblast activating protein (FAP) is a characteristic expression of cancer-related cells. [^68^Ga]FAPI-04 PET/CT demonstrates favorable performance in studying various solid tumors by labeling fibroblast activation protein inhibitors (FAPI) (11). Moreover, [^68^Ga]FAPI-04 PET/CT accumulates at a high level in tumors, showing excellent pharmacokinetic and biochemical characteristics (9). Compared with other imaging diagnosis methods, [^68^Ga]FAPI-04 PET/CT also demonstrated improved background signal levels (12). However, the expression of fibroblast activating protein (FAPI) is not significant in normal tissues, but it is highly expressed in abnormal conditions such as wound healing and arthritis, which affects the accuracy of [^68^Ga]FAPI-04 PET/CT to some extent (13–15).

In this paper, our objective is to conduct a meta-analysis by gathering available literature and its corresponding data, with the purpose of comparing the diagnostic performance of FAPI and FDG in the detection of bone metastases in malignant tumors. This analysis aims to provide an authoritative and comprehensive assessment of the two methods, while maintaining a cautious approach to drawing conclusions from the results.

## Materials and methods

### Search strategy

We endeavored to conduct a comprehensive search on the Pubmed, Embase, and Web of Science databases for relevant literature published between 2023 and 2025, utilizing the following term combinations: (1) positron emission tomography or PET, (2) FAPI-04 or ^68^Ga-FAPI or FAPI or fibroblast activation protein, and (3) bone metastasis or bone metastases.

### Inclusion and exclusion criteria

Inclusion criteria for relevant literature: 1. Studies involving patients with tumor bone metastasis. 2. Comparative studies directly comparing FDG and FAPI, which may be prospective or retrospective experimental studies. 3. Studies with a total sample size exceeding 10 individuals. 4. Sources of information with high credibility and comprehensive relevant data. Exclusion criteria: 1. Studies conducted on animals rather than humans. 2. Literature types such as abstracts, discussions, literature reviews, etc. 3. Studies lacking extractable relevant detection rates. Based on the aforementioned criteria, the titles and abstracts of articles were evaluated to determine their suitability for inclusion. Further assessment was conducted on the full texts of the retained articles to ensure compliance with the inclusion criteria.

### Quality assessment and data extraction

We utilized the Quality Assessment of Diagnostic Accuracy Studies (QUADAS-2) tool to independently assess the quality of the included studies. The risk of bias and applicability of each study were carefully evaluated by two researchers. The evaluation of each study was rated as high, low, or unclear in terms of risk of bias and applicability. To ensure the resolution of any potential disagreements, a third reviewer was engaged. The analysis was conducted using RevMan (version 5.3).

Data extraction from all included papers was independently performed by two researchers. The extracted data encompassed the following aspects: (1) Author and Publication Year;(2) Study Characteristics, including the author’s country, as well as relevant research design, analysis, and reference standard;(3) Patient Characteristics, encompassing clinical indications, the number of patients, whether chemotherapy was administered before PET, and the mean/median age;(4) Technical Characteristics, comprising imaging scanner modality, types of imaging tests, ligand dose, time interval between injection and acquisition, image analysis, and detection rate. When the information was not explicitly stated in the text, we attempted manual retrieval of data from the literature, tables, and figures. In instances where the paper lacked sufficient information, we reached out to the corresponding authors via email, requesting additional data or clarification. Any disagreements between the two researchers were resolved through mutual consensus.

## Results

### Study selection

**Figure 1:**
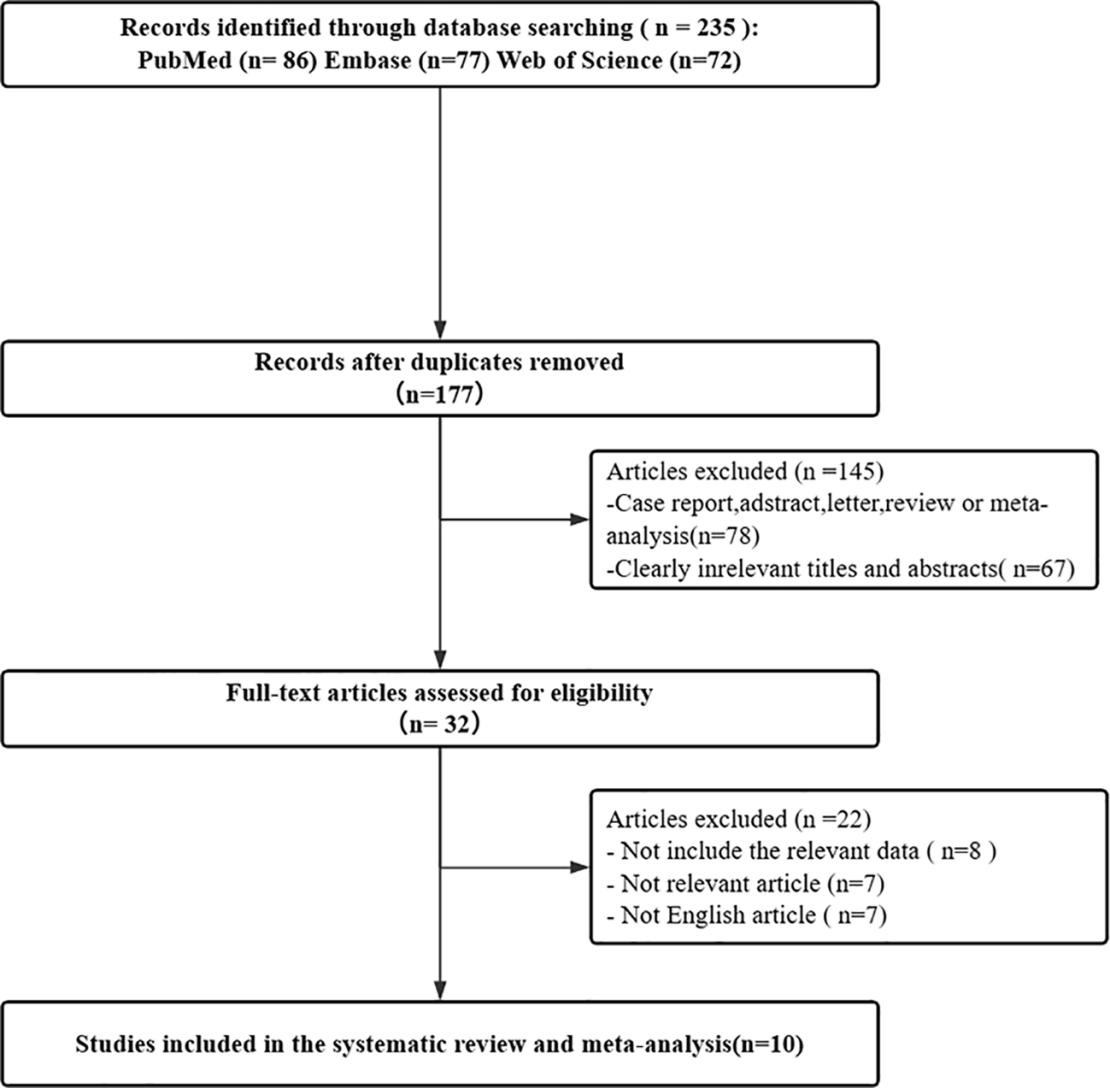
Flow diagram of study selection.

Initially, a total of 235 articles were identified in the search. After excluding 58 articles, 177 studies were determined as relevant. Among them, 78 case reports, abstracts, reviews, meta-analyses, and 67 articles with entirely unrelated titles and abstracts to this study were excluded, leaving 32 studies for further evaluation. Subsequently, 8 articles lacking relevant data, 7 articles irrelevant to the research, and 7 non-English articles were excluded, resulting in a total of 10 articles are related to the detection performance of [^68^Ga]FAPI-04 PET/CT and [^18^F]FDG PET/CT in malignant tumor bone metastasis (16–25 (p68)). The ProcessOn flow diagram of the study selection process is shown in **Figure 1**.

### Study description and quality assessment

**Figure 2:**
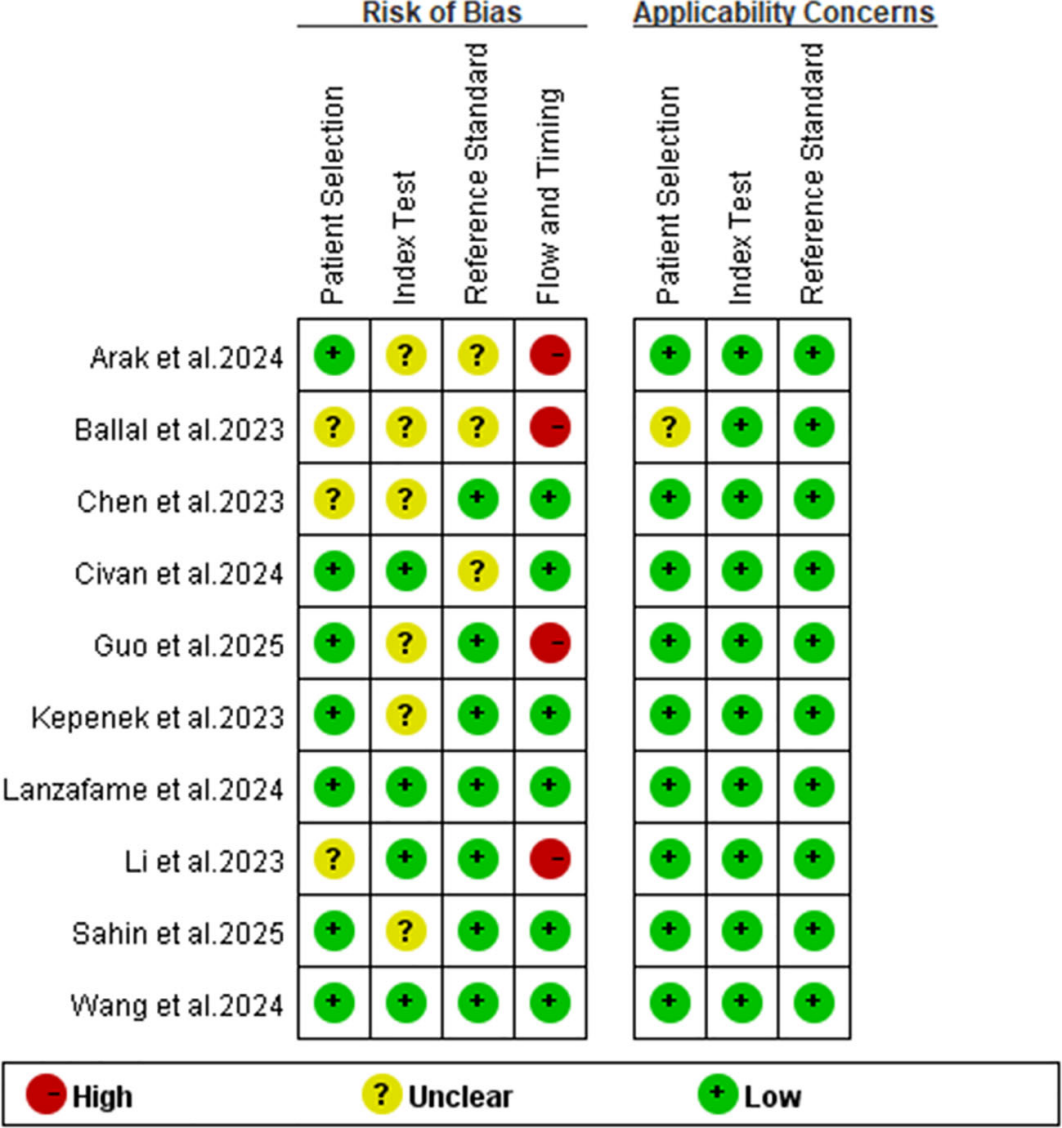
Summary of risk of bias and applicability concerns of the included studies.

**Table 1** provides an overview of the extracted research and patient characteristics from 10 different studies, which collectively involved 472 patients. Technical details are outlined in **Table 2**. Furthermore, an evaluation of the quality of the relevant studies was conducted using the Quality Assessment of Diagnostic Accuracy Studies (QUADAS-2) tool. The quality assessment plot indicates that the primary concentration of high-risk bias issues lies in the flow and timing domain((**Figure 2**), the possible reason for this could be the exclusion of some patients from the analysis in most studies. Overall, the bias risk in these articles was deemed satisfactory.

**Table 1:** Study and patient characteristics of the included studies.

Author	Year	Study characteristics			Patient characteristics			
		Country	Study design	Analysis	No. of patients	Clinical indication	Mean/Median age	Primary cancer
Sahin et al. (17)	2025	China	Pro	LB AND PB	23	Initial staging	Median =51 years	Lung Cancer
Wang et al. (18)	2024	China	Pro	LB AND PB	10	Initial staging Post-treatment	Median = 59 years	Gastric Carcinomas
Arak et al. (19)	2024	Turkey	Pro	LB	75	NA	Median = 55 years	NA
Guo et al. (20)	2025	China	Pro	LB AND PB	48	Initial staging Post-treatment	Median = 57 ± 13 years	NA
Civan et al. (21)	2024	Turkey	Pro	LB	20	Initial staging Post-treatment	Median = 62 years	Renal Carcinoma
Lanzafame et al. (16)	2024	German	Pro	LB	200	Initial staging Post-treatment	Median = 55 years	NA
Chen et al. (24)	2023	China	Pro	LB AND PB	34	Initial staging	Median = 51 years	Gastric Carcinomas
Ballal et al. (25)	2023	India	Pro	PB	5	NA	Median = 53.2 ± 11.7 years	Thyroid cancer
Kepenek et al. (22)	2023	Turkey	Pro	LB	44	Initial staging	Median = 56.82 ± 15.58 years	NA
Li et al. (23)	2023	China	Pro	LB	8	Initial staging Post-treatment	Median = 59.09 ± 10.98 years	Biliary tract carcinoma
Chen et al. (24)	2023	China	Pro	LB AND PB	5	Initial staging	Median = 51 years	Gastric Carcinomas

PB, patient-based; LB, lesion-based; Pro, prospective; Retro, retrospective; NA, not available.

**Table 2:** Technical aspects of included studies.

Author	Year	Scanner modality	Ligand dose ([68 Ga]Ga-DOTA-FAPI-04)	Time from injection to acquisition ([68 Ga]Ga-DOTA-FAPI-04)	Ligand dose ([18F]FDG)	Time from injection to acquisition ([18F]FDG)	Image analysis
Sahin et al. (17)	2025	GE Healthcare	2–3 MBq/kg	45min	5 MBq/kg	60min	Visual and quantitative
Wang et al. (18)	2024	Hebei Andike Positron Technology Co., Ltd	1.5–1.8MBq/kg	60 ± 5min	3.7 MBq/kg	60 ± 5min	Quantitative
Arak et al. (19)	2024	General Electric Company, Milwaukee, Wisconsin, USA and General Electric Company, Milwaukee, Wisconsin, USA	5 MBq/Kg	60min	2–3 MBq/kg	45min	Visual and quantitative
Guo et al. (20)	2025	Philips Medical Systems, The Netherlands and Philips Medical Systems, The Netherlands	3.7 MBq/kg	60 ± 10 min	1.9 to 3.7 MBq/kg	60 ± 10 min	Visual and quantitative
Civan et al. (21)	2024	GE Healthcare IQ Discovery PET/CT	125 MBq	15min	NA	NA	Quantitative
Lanzafame et al. (16)	2024	Siemens Healthineers	120 ± 38.3 MBq	23.5 ± 19.0 min	248.6 ± 89.2 MBq	69.5 ± 15.5 min	Visual and quantitative
Chen et al. (24)	2023	Discovery MI,G.E. Healthcare;BiograpmCT,Siemens Healthineers;	133.2–281.2MBq	60min	203.5–358.9MBq	60min	Visual and quantitative
Ballal et al. (25)	2023	NA	185–370 MBq	NA	185 - 370 MBq	NA	Quantitative
Kepenek et al. (22)	2023	GE Healthcare, Milwaukee, WI, USA	2 MBq/kg	NA	3.5–5.5 MBq/kg	NA	Visual and quantitative
Li et al. (23)	2023	Isotope Technologies Munich GmBH, Munich, Germany	1.85–3.7 MBq/kg	2.5min	1.85–3.7 MBq/kg	2min	Visual and quantitative
Chen et al. (24)	2023	NA	281.2 MBq (range, 203.5–358.9)	60min	194.3 MBq (range 133.2–281.2)	60min	Visual and quantitative

PB, patient-based; LB, lesion-based; Pro, prospective; Retro, retrospective; NA, not available.

### Diagnostic performance of [^68^Ga]FAPI-04 PET/CT and [^18^F]FDG PET/CT for bone metastasis

**Figure 3:**
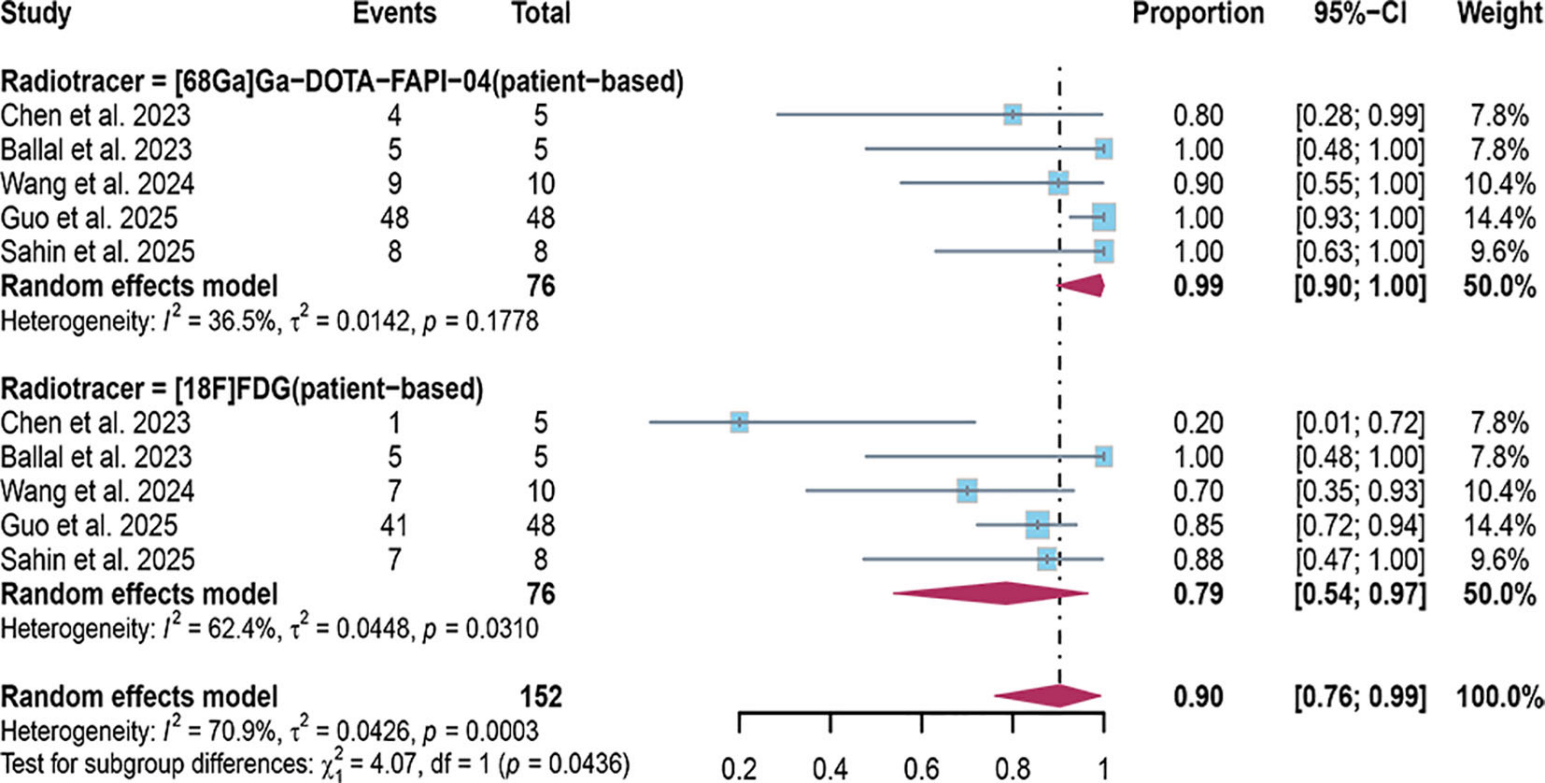
The forest plot shows the pooled detection rate of [^68^Ga]FAPI-04 PET/CT and [^18^F]FDG PET/CT in patient-based studies of bone metastases from malignant tumors. The symbol (◆) represents the estimated detection rate.

In the patient-based related studies, a random-effects model was utilized, and the results, as shown in **Figure 3**, indicate that the detection rate of [^68^Ga]FAPI-04 PET/CT was 0.99 (95% CI: 0.90–1.00, I²=36.5%), while that of [^18^F]FDG PET/CT was 0.79 (95% CI: 0.54–0.79, I²=62.4%). A statistically significant difference was observed between the two modalities (P = 0.0003).

**Figure 4:**
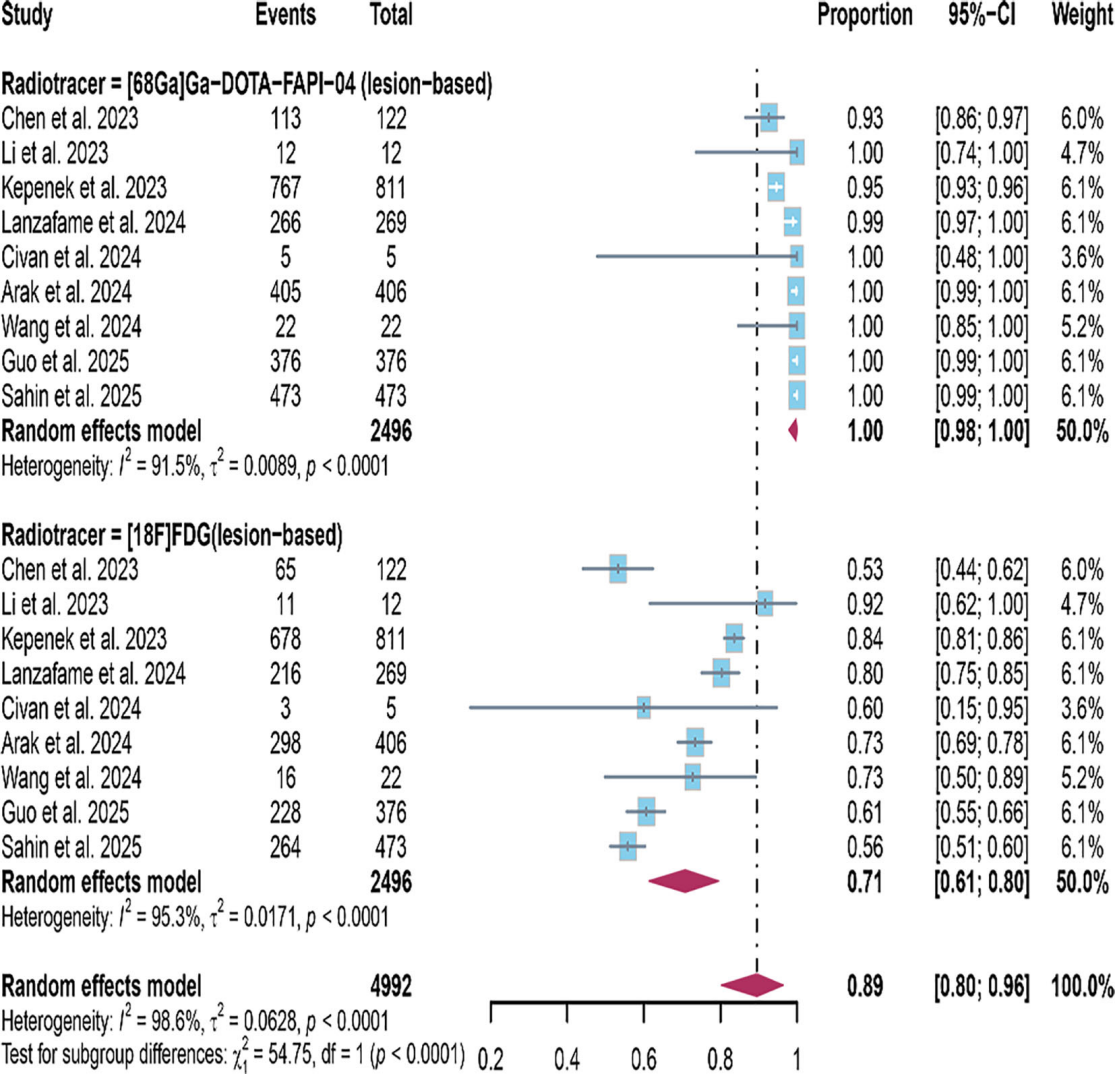
The forest plot shows the pooled detection rate of [^68^Ga]FAPI-04 PET/CT and [^18^F]FDG PET/CT in lesion-based studies of bone metastases from malignant tumors. The symbol (◆) represents the estimated detection rate.

In the lesion-based related studies, a random-effects model was also employed, and the results, presented in **Figure 4**, demonstrate that the detection rate of [^68^Ga]FAPI-04 PET/CT was 1.00 (95% CI: 0.98–1.00, I²=91.5%), whereas [^18^F]FDG PET/CT showed a detection rate of 0.71 (95% CI: 0.61–0.80, I²=95.3%). The difference in detection rates between the two imaging methods was statistically significant (P<0.0001).

### Heterogeneity analysis

**Figure 5:**
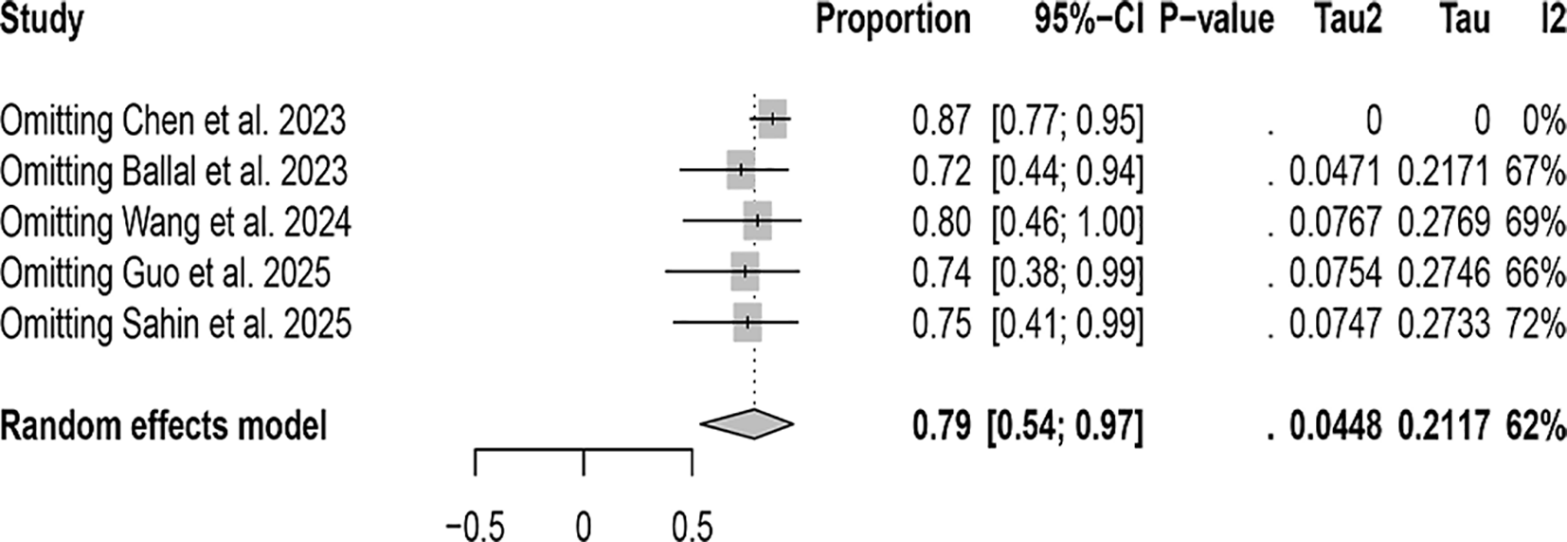
Sensitivity analysis of patient-based detection using [¹⁸F]FDG PET/CT.

In the patient-based studies, the detection rate of [^68^Ga]FAPI-04 PET/CT was 0.99 (95% CI: 0.90–1.00, I²=36.5%), indicating no significant heterogeneity among the included studies (I² < 50%). In contrast, the detection rate of [^18^F]FDG PET/CT was 0.79 (95% CI: 0.54–0.79, I²=62.4%). Sensitivity analysis suggested that the study by Chen et al. might be the primary source of heterogeneity; after its exclusion, the heterogeneity decreased to I²=0(**Figure 5**).

**Figure 6:**
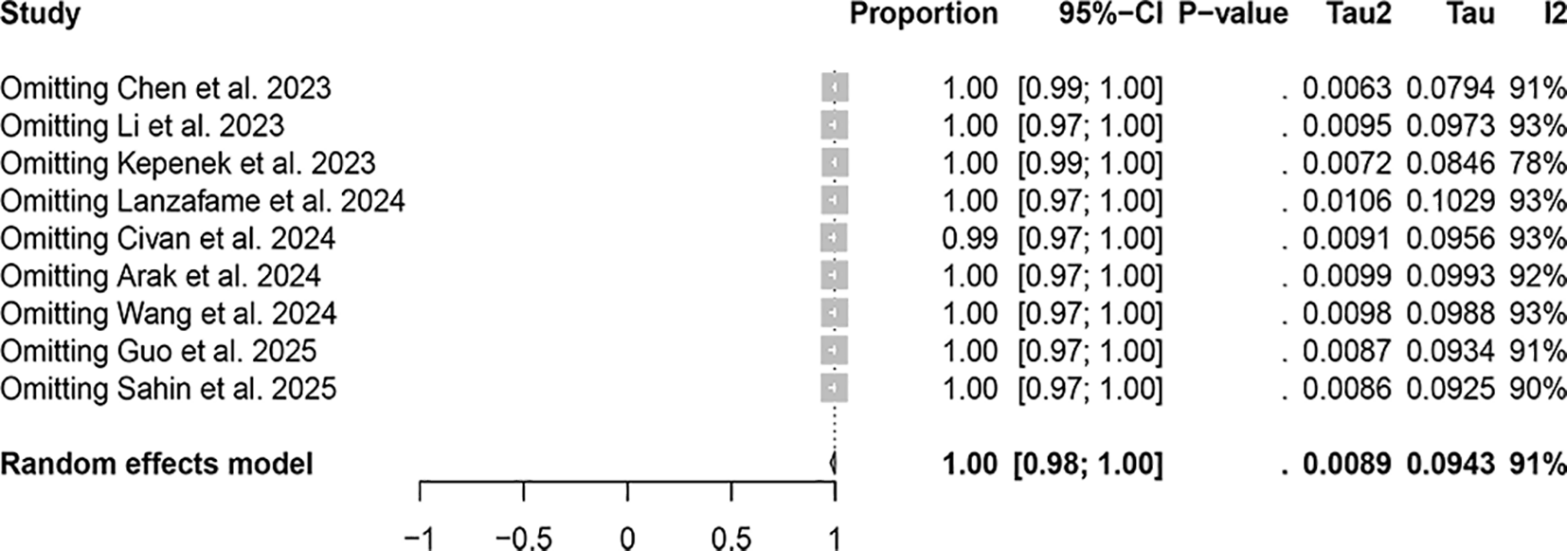
Sensitivity analysis of lesion-based detection using [⁶⁸Ga]FAPI-04 PET/CT.

**Figure 7:**
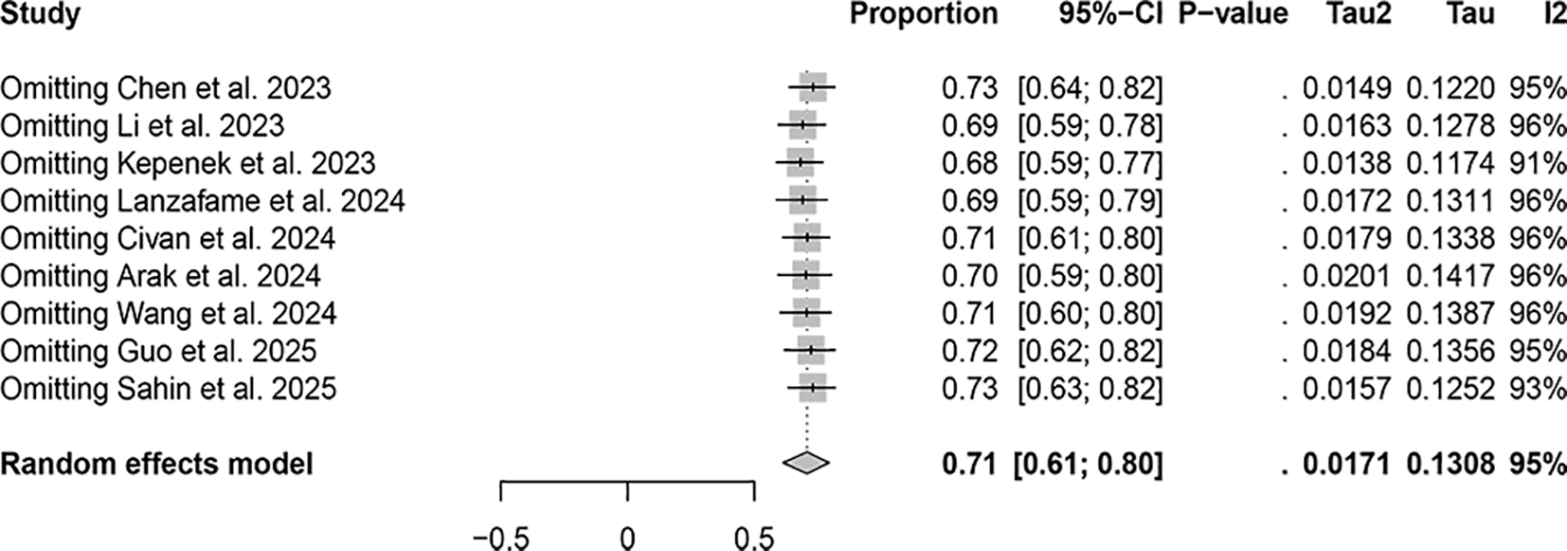
Sensitivity analysis of lesion-based detection using [¹⁸F]FDG PET/CT.

The lesion-based studies, [^68^Ga]FAPI-04 PET/CT was 1.00 (95% CI: 0.98–1.00, I²=91.5%), whereas that of [18F]FDG PET/CT was 0.71 (95% CI: 0.61–0.80, I²=95.3%). Sensitivity analysis did not identify any apparent sources of heterogeneity (**Figures 6**, **7**).

## Discussion

The diagnostic performance of [^68^Ga]FAPI-04 PET/CT and [^18^F]FDG PET/CT in detecting bone metastases from malignant tumors remains a subject of ongoing investigation, as current findings show notable inconsistencies across studies. For example, Yue et al. reported that [^68^Ga]FAPI-04 PET/CT demonstrated superior sensitivity compared with [^18^F]FDG PET/CT in detecting tumor recurrence and peritoneal lymph node metastases, suggesting potential advantages in certain metastatic patterns. However, [^18^F]FDG PET/CT exhibited higher sensitivity in identifying bone metastases (26).Similarly, Dna et al. found that in the assessment of bone metastases from malignant tumors, [^68^Ga]FAPI-04 PET/CT showed slightly lower pooled sensitivity than [^18^F]FDG PET/CT in patient-based analyses, whereas [^18^F]FDG PET/CT demonstrated higher sensitivity in lesion-based analyses (27). This difference may arise from the distinct biological mechanisms reflected by the two tracers: FDG indicates tumor glucose metabolism, while FAPI reflects the activation of cancer-associated fibroblasts, representing tumor cells and the tumor microenvironment, respectively.

Meanwhile, FAPI also shows increased uptake in several benign skeletal conditions—such as osteoarthritis, fracture healing, fibrosis or post-radiotherapy changes, osteomyelitis, and postoperative or mechanical stress reactions—which may interfere with lesion-based evaluation and lead to overestimation of its diagnostic performance (15, 28). Additionally, differences in uptake timing between the tracers, particularly the rapid clearance and early imaging characteristics of FAPI, may influence image quality and quantitative assessment. Considering the variability in metabolic activity and stromal activation across different tumor types and clinical contexts, the comparative diagnostic value of [^68^Ga]FAPI-04 PET/CT and [^18^F]FDG PET/CT remains insufficiently defined. Thus, larger and methodologically robust prospective studies are needed to clarify their roles in detecting bone metastases.

Regarding the detection rates in patients with malignant tumor bone metastasis, [^68^Ga]FAPI-04 PET/CT demonstrates a detection rate of 0.99 (95% CI: 0.90–1.00, I²=36.5%), while [^18^F]FDG PET/CT was 0.79 (95% CI: 0.54–0.79, I²=62.4%).Regarding the detection rates of bone metastatic lesions, [^68^Ga]FAPI-04 PET/CT exhibits a detection rate of 1.00 (95% CI: 0.98–1.00, I²=91.5%), whereas [^18^F]FDG PET/CT demonstrates a detection rate of 0.71 (95% CI: 0.61–0.80, I²=95.3%). These results suggest that [^68^Ga]FAPI-04 PET/CT appears to have superior diagnostic capabilities compared to [^18^F]FDG PET/CT, whether in detecting bone metastasis in patients or detecting bone metastatic lesions. This result is consistent with the findings reported by Li et al. and Wu et al. (29, 30).The possible explanation for this observation is that [^68^Ga]FAPI and [^18^F]FDG reflect two different aspects of tumor behavior. [^18^F]FDG directly targets the metabolic activity of tumor cells, whereas [^68^Ga]FAPI targets fibroblast activation protein expressed on cancer-associated fibroblasts within the tumor stroma (31, 32). FAP is a marker of desmoplastic reaction and has been reported to play a key role in influencing tumor immunity and multidrug resistance, which may be related to reduced transtumoral transport of cells and therapeutic agents (33).

In the patient-based studies, the I² value for [^68^Ga]FAPI-04 PET/CT was less than 50%, indicating no significant heterogeneity among the included studies. In contrast, for [^18^F]FDG PET/CT, the heterogeneity markedly decreased to I² = 0 after excluding the study by Chen et al., suggesting that this study may have been the primary source of heterogeneity. In the lesion-based studies, sensitivity analysis did not reveal any apparent sources of heterogeneity. We believe that large-scale, multicenter prospective studies are still needed, with standardized study designs and imaging protocols, to minimize heterogeneity as much as possible. In addition, subgroup analyses based on tumor type, metastatic burden, and imaging equipment could help to further identify the potential sources of heterogeneity, thereby improving the robustness and reliability of the pooled results. Such efforts would also help to further validate and clarify the differences and advantages between [^68^Ga]FAPI-04 PET/CT and [^18^F]FDG PET/CT in the diagnosis of bone metastases.

Our meta-analysis also has certain limitations. First, this study mainly focused on comparing the detection rates of [^68^Ga]FAPI-04 PET/CT and [^18^F]FDG PET/CT, without extracting data on false positives, false negatives, or other diagnostic parameters. Given that FAPI may show increased uptake in inflammatory or other non-malignant conditions (13, 14), this could influence the results. Second, the sample size was relatively small, and larger datasets are required to strengthen the conclusions. Moreover, although substantial heterogeneity was observed in both lesion-based and patient-based analyses, no subgroup or stratified analyses were actually performed to explore potential sources of heterogeneity related to [^18^F]FDG PET/CT. Therefore, these limitations should be considered when interpreting the findings.

## Conclusion

In the context of malignant bone metastases, both patient-based and lesion-based analyses suggest that [^68^Ga]FAPI-04 PET/CT may exhibit higher detection rates than [^18^F]FDG PET/CT. However, increased FAPI uptake can also occur in inflammatory or other benign conditions, which may influence these findings. Moreover, the available PET/CT evidence is derived from studies with relatively small sample sizes. Importantly, detection rate alone cannot distinguish true-positive from false-positive findings. Therefore, larger and methodologically rigorous prospective studies are required to more clearly define the comparative diagnostic performance of these tracers.

## Data Availability

The original contributions presented in the study are included in the article/supplementary material. Further inquiries can be directed to the corresponding author.
